# CASE: a framework for computer supported outbreak detection

**DOI:** 10.1186/1472-6947-10-14

**Published:** 2010-03-12

**Authors:** Baki Cakici, Kenneth Hebing, Maria Grünewald, Paul Saretok, Anette Hulth

**Affiliations:** 1 Swedish Institute for Infectious Disease Control (SMI), 171 82 Solna, Sweden; 2 Royal Institute of Technology (KTH), 100 44, Stockholm, Sweden

## Abstract

**Background:**

In *computer supported outbreak detection*, a statistical method is applied to a collection of cases to detect any excess cases for a particular disease. Whether a detected aberration is a true outbreak is decided by a human expert. We present a technical framework designed and implemented at the Swedish Institute for Infectious Disease Control for computer supported outbreak detection, where a database of case reports for a large number of infectious diseases can be processed using one or more statistical methods selected by the user.

**Results:**

Based on case information, such as diagnosis and date, different statistical algorithms for detecting outbreaks can be applied, both on the disease level and the subtype level. The parameter settings for the algorithms can be configured independently for different diagnoses using the provided graphical interface. Input generators and output parsers are also provided for all supported algorithms. If an outbreak signal is detected, an email notification is sent to the persons listed as receivers for that particular disease.

**Conclusions:**

The framework is available as open source software, licensed under GNU General Public License Version 3. By making the code open source, we wish to encourage others to contribute to the future development of computer supported outbreak detection systems, and in particular to the development of the CASE framework.

## Background

In this paper, we describe the design and implementation of a *computer supported outbreak detection system *called CASE (named after the protagonist of the William Gibson novel Neuromancer), or Computer Assisted Search for Epidemics. The system is currently in use at the Swedish Institute for Infectious Disease Control (SMI) and performs daily surveillance using data obtained from *SmiNet *[1], the national notifiable disease database in Sweden.

Computer supported outbreak detection is performed in two steps:

1 A statistical method is automatically applied to a collection of case reports in order to detect an unusual or unexpected number of cases for a particular disease.

2 An investigation by a human expert (an epidemiologist) is performed to determine whether the detected irregularity denotes an actual outbreak.

The main function of a computer supported outbreak detection system is to warn for *potential *outbreaks. In some cases, the system might be able to detect outbreaks earlier than human experts. Additionally, it might detect certain outbreaks that human experts would have overlooked. However, the system does not aim to replace human experts (hence the prefix "computer supported"); it should rather be considered a complement to daily surveillance activities. To a smaller extent, the system can also aid less experienced epidemiologists in identifying outbreaks.

Systems for outbreak detection which support multiple algorithms include RODS [2], BioSTORM [3] and AEGIS [4]. Additionally, computer supported outbreak detection systems operating on the national level have been used previously in a number of countries, including Germany [5] and the Netherlands [6].

### Health care in Sweden

The health care system in Sweden is governed by 21 county councils. Each county has appointed a medical officer, who is in charge of the regional infectious disease prevention and control. Every confirmed or suspected case of a notifiable disease is reported both to the county medical officer and to SMI. At SMI, the regular national surveillance is currently performed by thirteen epidemiologists, each in charge of a number of different diseases.

All 21 county medical officers as well as the majority of the hospitals and the laboratories in Sweden are connected to the SmiNet database. The database collects clinical reports and information on laboratory verified samples. In 2008, a total of 174 811 reports were submitted to SmiNet. 87 per cent of these reports were submitted electronically and those that were not submitted electronically were entered into SmiNet manually. Of the 92 744 lab reports, as much as 97 per cent were submitted electronically and 62 per cent fully automatically. The reports were subsequently merged into 74 367 case reports. These reports form the basis of the data used by CASE to perform outbreak detection.

## Implementation

CASE is designed to be administered using a graphical interface, and can operate on all of the 63 notifiable diseases in Sweden. One or more statistical detection methods can be applied to each disease. If more than one method is activated, result reports are generated independently. By default, the data are aggregated over all disease subtypes, but the system allows detection of single subtypes as well. When an outbreak signal is generated, an alert is sent by email to all members of the notification list for that particular disease.

CASE is composed of three interconnected components for *configuration*, *extraction *and *detection*. The configuration component provides a graphical user interface for modifying detection parameters and editing the list of recipients for generated alerts. The extraction component is used to copy data from the national case database to the local database. The detection component is scheduled to run at regular intervals and automatically applies the chosen statistical methods to the currently selected diseases.

### System Description

CASE is developed using Java to ensure platform-independence of all components. Currently at SMI all three components run on Ubuntu, a Linux-based operating system. The local database for CASE is MySQL and the national database, SmiNet, is Microsoft SQL Server 2005.

**Figure 1:**
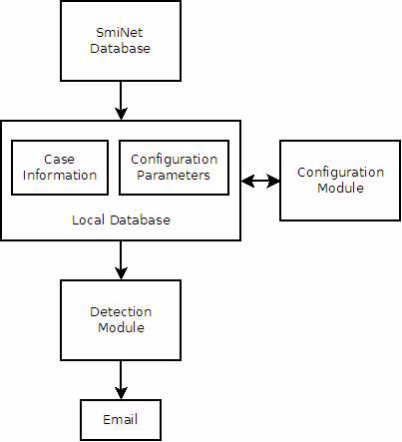
**CASE Flowchart**. A flowchart demonstrating the detection process in CASE.

Figure 1 shows the flow of information within the framework. The extraction and detection components are scheduled to run once every 24 hours at midnight using the standard Unix scheduling service *cron*. When the extraction component is executed, it transfers data from SmiNet to the local database. The local database stores the case data and the configuration parameters for all algorithms. The configuration module can be used to view and modify the parameters. The detection component is executed automatically after all required data have been extracted from SmiNet. It applies the detection methods with the given parameters to the case data for the selected diseases, and emails notifications if any alerts are generated. Detailed logs of these processes are generated automatically.

#### Configuration

The configuration component is a graphical user interface that allows the administrator to mark diseases for detection, choose the detection methods to be applied to each diagnosis/subtype and manage the list of epidemiologists that will receive alerts in case a warning is generated. The settings are stored in a local database that is also accessed by the other two components. The system can be administered by multiple users who access the same local database.

**Figure 2:**
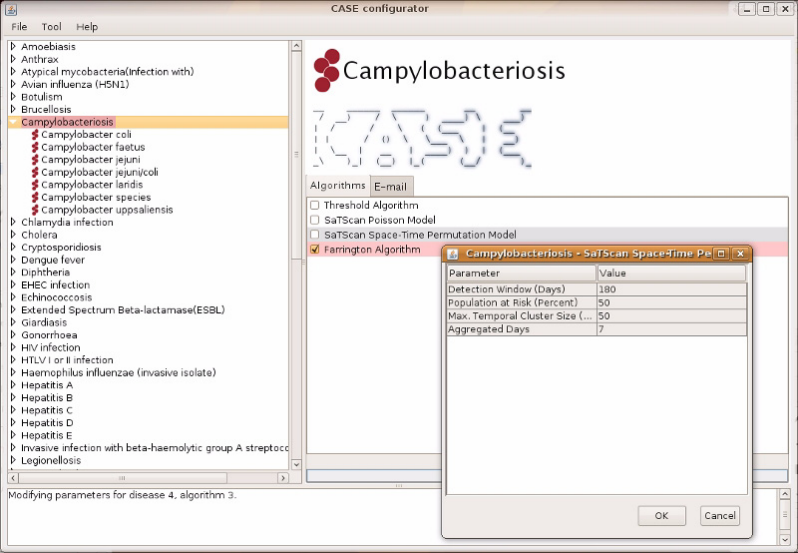
**Administrator GUI**. A screenshot of the graphical user interface for the CASE administrator.

Figure 2 shows a screenshot of the graphical user interface for the CASE administrator. The notifiable diseases are displayed in the left column. These entries can be expanded using the arrow to display their subtypes. Parameters for the current selection are shown on the right hand side. The *Algorithms *tab lists the available methods. Parameters for the selected method can be modified by double-clicking the name of the method. The *E-mail *tab contains a list of recipients for the selected disease and/or subtype. If an alert is generated after detection, the algorithm that generated the alert is highlighted in red. The flag is automatically cleared every night before a new detection batch is executed.

#### Extraction

CASE uses data retrieved from SmiNet to perform outbreak detection. A case report is created in SmiNet when a clinical or a laboratory report is received, provided that this patient does not already exist in the database. When additional reports arrive, the original case report is automatically updated with the new information. Depending on the number of days that have elapsed since the last time a patient received a particular diagnosis, a new case report might be created for the same diagnosis and patient. For a detailed technical description of SmiNet, see [1].

The extraction component populates the local database with data from the case reports stored in SmiNet. Diagnosis, lab species, date, and reporting county are copied for every case, except those with infections that are reported to have originated abroad. No information that can reveal a patient's identity is used in the outbreak detection process. There are approximately twenty dates in SmiNet for each case report, ranging from dates that are automatically generated by the system to dates entered by the clinician or the laboratory. There is, however, only one date that is available on all case reports, namely *statistics date*. This automatically set date corresponds to when a patient first appears in SmiNet with a particular diagnosis. The date that would best reflect when a patient fell ill is the date when the sample was taken from the patient. However, many case reports do not contain this date. For example, for 2008 this date is missing in 29 per cent of the case reports. When the case information is copied from SmiNet to the local database, the extraction component fetches the statistics date as the date for the case.

#### Detection

CASE is developed by the Swedish Institute for Infectious Disease Control, and has a national perspective on outbreaks. Its primary role is to find outbreaks that cover more than one county, especially those with few cases in each affected county, as these might be difficult to detect for the local authorities.

The detection component uses the selected statistical method(s) on all activated diseases and sends notification emails if any alerts are raised. If there are too few data points for a detection algorithm to produce a result -- which is often the case for detection on the subtype level -- this information is written to the log file. The system currently supports four different statistical methods for detection: SaTScan Poisson [7], SaTScan Space-Time Permutation [8], an algorithm developed by Farrington et al. [9], and a simple threshold algorithm. The methods are briefly described below. Three of the four methods are freely available implementations, while the fourth was developed within the project and is included in CASE's source code. For the external programs, input generators and output parsers are also contained within the source code. It is possible to extend the system with additional statistical methods, although this requires a certain familiarity with the Java programming language. We are currently in the process of adding the *OutbreakP *method [10] to the core package.

SaTScan is a freely available spatial, temporal and space-time data analysis platform [11]. Two algorithms from this application are used in CASE: *SaTScan Poisson *which uses the discrete Poisson SaTScan model to search for spatial clusters and *SaTScan Space-Time Permutation*, which searches for spatio-temporal clusters. Both models are applied to data at the county-level resolution. The population data required by SaTScan Poisson are obtained from Statistics Sweden [12]. The SaTScan Poisson parser, developed specifically for CASE, raises an alert if a detected cluster ends within the last week.

The third detection method was developed by, and is in regular use at the Health Protection Agency in England and Wales [9]. In CASE, we use the surveillance R-package implementation [13] of the method and we refer to it as the *Farrington algorithm*. The algorithm is used on data aggregated at the national level, to investigate if the current disease incidence exceeds that of the reference data from previous years. The CASE parser for the Farrington output ensures that an alert is sent only if an exceedance occurred during the last two weeks. The required window size is implemented as a sliding window of seven days and detection is performed daily.

The *threshold algorithm *is used to generate alerts when the number of cases for a particular disease rises above a manually defined value, with the number of cases aggregated at the national level.

**Figure 3:**
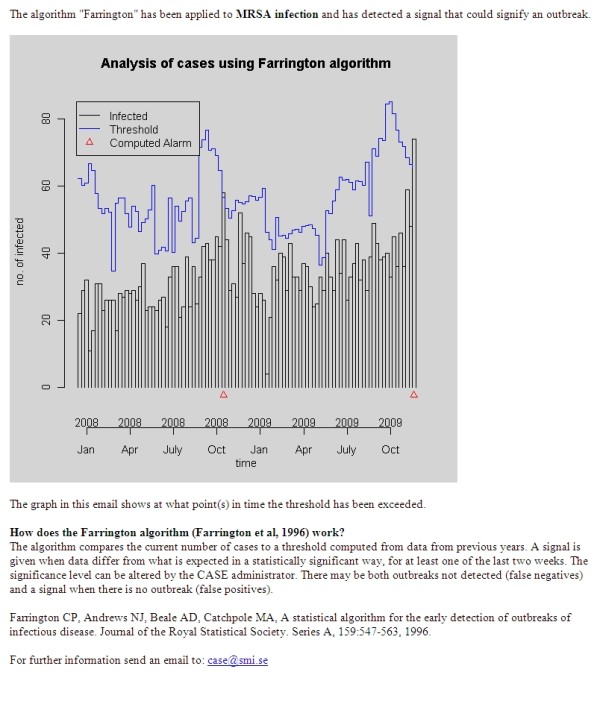
**Alert Email**. A sample email for a disease alert.

For all methods, as long as an outbreak is ongoing according to the results of the statistical analysis, a new alert is raised every night. Figure 3 shows an alert email that is sent to the recipients of "MRSA infection". The graph is automatically generated by the detection component and shows all computed alarms on the x-axis. The computed threshold is denoted by the blue curve (the graph in Figure 3 was generated using simulated data). The email also includes a brief description of the algorithm that generated the alarm.

## Results and Discussion

CASE is a technical framework designed to ease the process of connecting a data source with reported cases to various statistical methods requiring different input formats. When using CASE, the user can select the methods that are best suited to the characteristics of a particular disease.

CASE can also be used as a platform for comparing different detection algorithms, although that is not its primary purpose. Since all algorithms use the same data, running multiple detection methods on the same disease regularly and comparing the successful detections and the false warnings can provide insights into the accuracy of a certain method for a given disease. Comparisons and evaluations of the statistical methods currently included in CASE can be found in, for example, [14] and [15]. Here, the importance of calibrating the parameters for the detection methods must be emphasized, something which is still an ongoing work at SMI.

At present, the evaluation of the system is mainly qualitative, consisting of frequent discussions between the epidemiologists and the CASE developers. There is, however, a need for more systematic evaluations of the system, including a questionnaire assessing the users' experience, in addition to quantitative evaluations of the performance of the algorithms and the parameter settings. To facilitate the quantitative evaluations, we plan to extend the functionality of CASE to incorporate an evaluation module allowing the algorithms to be run retrospectively, with analysis carried out for each day in a specified time period. The main objective is not a general comparison of the algorithms, but an assessment of their performance in the specific context of the data they are used on. Where external data telling when actual outbreaks have occurred are available, measures such as sensitivity and specificity can be calculated. The evaluation module would provide valuable guidance in the choice of algorithms and parameter settings for the end user. Another evaluation feature we consider implementing is the possibility to run simulated data in the system.

CASE currently uses emails for notification. The advantage of this approach is that it presents information to the users in a familiar way and does not require them to learn how to operate a new interface. The disadvantage, on the other hand, is that the system becomes one-sided if the emails do not include a feedback mechanism. Regardless of the actual implementation, a system for providing feedback from the receivers of the signals is essential. Currently, users who would like to provide feedback on CASE output are instructed to email the administrator.

As expected, a relatively simple method operating on accurate and informative data produces better results than a complex method operating on noisy data. Therefore, the most important factor for creating a reliable outbreak detection system is to ensure the quality of the input data. If the input is not reliable, improving the data collection process from local medical centres is a much better investment than trying to perform automatic detection on inaccurate data. Additionally, expectations from an automated detection system must be realistic. For a computer, detecting ongoing outbreaks and seasonal regular outbreaks is possible, but predicting an outbreak at onset is currently not feasible.

CASE is designed primarily to analyze case reports and does not provide syndromic surveillance support using external data sources, unlike RODS [2] or BioSTORM [3]. The only requirement for the operation of CASE is access to a case database for notifiable diseases. All scripts to create and configure the intermediate local database are included in the software package. The local database is used to selectively copy and store case reports after removing all information that can reveal a patient's identity. We believe that the ease of configuration and maintenance in addition to the possibility of operating without storing highly sensitive data make CASE a strong candidate for use in national infectious disease surveillance.

## Conclusions

In this paper we have described the design and implementation of a publicly available technical framework for computer supported outbreak detection. The source code is licensed under GNU GPLv3 [16] and is available from https://smisvn.smi.se/case.

The CASE framework is designed to be a complete system for computer supported outbreak detection at the national level. We are aware that any outbreak detection system must always be adapted to a particular context, where national requirements and regulations will affect the implementation of the system. Such adaptations can easily be made within the described framework. By making the code open source, we wish to encourage others to contribute to the future development of computer supported outbreak detection systems, and in particular to the development of the CASE framework.

## Availability and requirements

The source code for CASE is licensed under GNU General Public License Version 3 (GPLv3), and is available for download from https://smisvn.smi.se/case. The provided documentation and the interface are written in English. The following software must be installed on the target system in order to use CASE:

• Linux or Windows operating system that can run Sun Java Runtime Environment 6.0 (or higher)

• MySQL 5.1 (or higher)

• SaTScan version 8.0.1 (or higher)

• R version 2.9.1 (or higher)

• ImageMagick 6.5.4 (or higher)

## Competing interests

The authors declare that they have no competing interests.

## Authors' contributions

AH, BC and PS designed and developed the CASE framework. BC, KH and PS implemented the framework. KH and MG worked on improving the application. AH and BC drafted the manuscript. All authors read and approved the final manuscript.

## Pre-publication history

The pre-publication history for this paper can be accessed here:

http://www.biomedcentral.com/1472-6947/10/14/prepub
